# Hydrogels for Biomedical Applications: Their Characteristics and the Mechanisms behind Them

**DOI:** 10.3390/gels3010006

**Published:** 2017-01-24

**Authors:** Qinyuan Chai, Yang Jiao, Xinjun Yu

**Affiliations:** Department of Chemistry, University of Cincinnati, Cincinnati, OH 45221, USA; chaiqn@mail.uc.edu (Q.C.); jiaoyn@mail.uc.edu (Y.J.)

**Keywords:** hydrogels, biomaterials, drug delivery, self-healing, cell culture

## Abstract

Hydrogels are hydrophilic, three-dimensional networks that are able to absorb large quantities of water or biological fluids, and thus have the potential to be used as prime candidates for biosensors, drug delivery vectors, and carriers or matrices for cells in tissue engineering. In this critical review article, advantages of the hydrogels that overcome the limitations from other types of biomaterials will be discussed. Hydrogels, depending on their chemical composition, are responsive to various stimuli including heating, pH, light, and chemicals. Two swelling mechanisms will be discussed to give a detailed understanding of how the structure parameters affect swelling properties, followed by the gelation mechanism and mesh size calculation. Hydrogels prepared from natural materials such as polysaccharides and polypeptides, along with different types of synthetic hydrogels from the recent reported literature, will be discussed in detail. Finally, attention will be given to biomedical applications of different kinds of hydrogels including cell culture, self-healing, and drug delivery.

## 1. Introduction

Hydrogels, crosslinked 3D networks of hydrophilic polymer chains, are capable of holding large amounts of water due to their hydrophilic structure [1,2,3,4]. Thus, the hydrogel networks can extensively swell in water media. Since water is the greatest component of the human body, a hydrogel, which can absorb large quantities of water, is considered to have great potential when applied for biomedical purposes [5,6,7,8,9,10]. Recently, wide investigation has been going on into the feasibility of applying hydrogels in fields including tissue engineering, drug delivery, self-healing materials, biosensors, and hemostasis bandages [11,12,13,14,15]. Compared with other types of biomaterials, hydrogels have the advantages of increased biocompatibility, tunable biodegradability, properly mechanical strength, porous structure, and so on. However, due to the low mechanical strength and fragile nature of the hydrogels, the feasibility of applying hydrogels is still limited. Thus, novel hydrogels with stronger and more stable properties are still needed and remain an important direction for research.

As expected, naturally formed hydrogels are gradually replaced by synthetic hydrogels to achieve longer service life, high capacity of water absorption, and high gel strength [8,9]. Fortunately, with various developed synthetic strategies, hydrogels with defined network structures, desirable chemical compositions, and tunable mechanical strength can be designed. Hydrogels can be prepared from completely artificial components and show remarkable stability even under severe conditions such as high temperature or a very acidic or basic environment. Additionally, by modifying the polymer chains with stimuli-responsive functional groups, the hydrogel properties can be switched by stimuli including heat, light, magnetic fields, chemical agents, and pH [16,17,18,19,20].

In this critical review, different technologies for preparing hydrogels will be discussed and we will take a closer look at different kinds of stimuli-responsive hydrogels. Detailed swelling mechanisms proposed based on various theories will be covered to give a deeper understanding of such materials. Last but not least, attention will be given to different hydrogels to achieve specific requirements for biomedical purposes such as cell culture, self-healing, and drug delivery.

## 2. Different Kinds of Stimuli-Responsive Hydrogels

Defined by Peppas [21], “hydrogels are hydrophilic, three-dimension networks, which are able to imbibe large amounts of water or biological fluids, and thus resemble, to a large extent, a biological tissue”. They are insoluble in any solvent due to the polymer chains being crosslinked by either covalent bonds or physical interactions such as entanglements and crystallites [22,23,24]. Due to the properties of hydrogels, such as high content of water, soft and rubbery consistence, as well as low interfacial tension with water or biological fluids, they are expected to be potential alternatives for natural tissues [25]. According to different applications, the hydrogel can be prepared to respond to various stimuli in the body such as pH, ionic strength, and temperature.

### 2.1. Thermoresponsive Hydrogels

The equilibrium between the hydrophobic and hydrophilic segments is the key to control the properties of a synthetic thermoresponsive hydrogel. In detail, the temperature has a remarkable effect on the hydrophobic interactions between hydrophobic polymer segments and the hydrophilic interactions between hydrophilic polymer segments and water molecules. Thus, a small temperature change can interrupt the original equilibrium and induce sol–gel transition [26]. In the work done by Vernon’s group [27], they synthesized a temperature-responsive graft copolymer based on *N*-isopropylacrylamide (NIPAAm) and Jeffamine M-1000 acrylamide (JAAm), which showed controlled swelling properties without introducing degradable moieties or increasing the lower critical solution temperature (LCST) above body temperature. The JAAm was hypothesized to be useful as a component in the polymer architecture to control the swelling and release properties with minimal effect on LCST. The outstanding hydrolytic stability, hydrophilicity, and minimal LCST effect make this hydrogel a suitable candidate for a variety of temperature-responsive biomaterials where control over swelling or drug release is crucial.

In another work [28], Long and co-workers investigated the application of a poly(*N*-isopropylacrylmide) (PNIPAAm) hydrogel thin film to thermochromic smart windows. The solar modulating ability (ΔT_sol_) showed an ultra-large value with the luminous transmittance (T_lum_) to be highest identified so far. This hydrogel demonstrated an excellent combination of high T_lum_ along with dramatically improved solar modulating ability, which may lead to the development of thermochromic smart windows based on organic materials.

### 2.2. pH-Responsive Hydrogels

pH-Responsive hydrogels are a class of biomaterials that exhibit desirable physical and chemical properties at specific pH ranges. Acidic or basic groups are bonded to the polymer chains. The acidic groups deprotonate at high pH, while the basic groups protonate at low pH. The association, dissociation, and binding of various ions to polymer chains cause hydrogel swelling in an aqueous solution.

Peppa’s group fabricated a hydrophilic pH-responsive hydrogel based on poly(methyacrylic-*graft*-ethylene glycol) (P(MMA-*g*-EG)) conjugated with hydrophobic PMMA nanoparticles [29]. By incorporating a different mole ratio of PMMA nanoparticles in the P(MMA-*g*-EG), it forms amphiphilic polymer carriers with tunable physical properties. The release of encapsulated therapeutic agents triggered by a change of pH from the stomach to the small intestine was tested. Furthermore, the cyto-compatibility of the polymer materials was investigated on cells modeling the gastrointestinal (GI) tract and colon cancer cells. This pH-responsive nanoparticle containing P(MMA-*g*-EG) provides the possibility to be used as oral delivery vectors of chemotherapeutics for cancer.

In general, the size of the gel will respond to environment pH as well as salt concentration. Thus, an equilibrium model was established by Moore’s group to predict the swelling/deswelling behavior of hydrogels in different pH solutions. The validation of the model was conducted by comparing the simulations with experimental results. This model was then utilized to investigate the effects of different hydrogels and solution conditions on the degree and rate of swelling/deswelling of those hydrogels [30]. It was found that the higher the concentration and buffer diffusivity are, the faster the kinetic. All these parameters can be used to tune the performance of hydrogel microactuators, suggesting that the mechanical properties of the hydrogel can be varied considerably by varying the pH of solutions.

### 2.3. Light- and Chemical-Responsive Hydrogels

**Figure 1:**
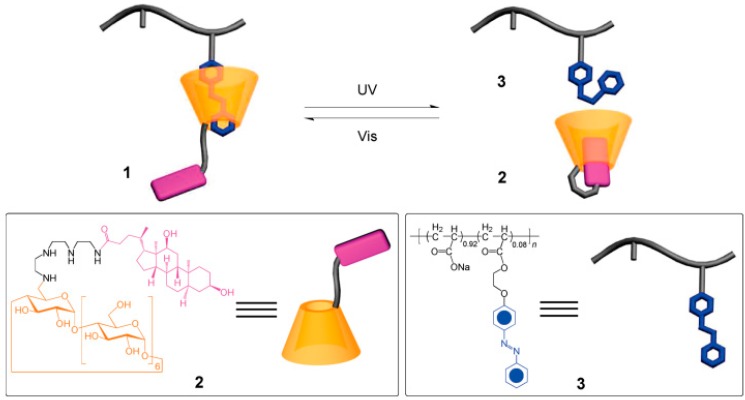
Supramolecular inclusion complex **1** formed from deoxycholate-β-CD derivative **2** and azobenzene-branched poly(acrylic acid) copolymer **3**. Reprinted from [34] with permission from the American Chemical Society (2009).

Light-responsive hydrogels are promising functional materials for potential application in the areas of drug/gene delivery [31], micro lenses [32], sensors [33], etc. due to the fact that the activation process via light can be remote and noninvasive. The prepared hydrogel consists of a deoxycholic acid-modified β-cyclodextrin derivative and an azobenzen-branched poly(acrylic acid) copolymer, and can be converted efficiently from gel to sol phase upon photo irradiation with light of 355 nm. The hydrogel was able to recover from sol to gel phase upon photo irradiation with light of 450 nm (Figure 1) [34]. The reversible transition of this hydrogel can be controlled under mild condition, suggesting that this gel material has a promising role in bioengineering applications for the release of molecular and cellular species.

In another example, a novel light-responsive hydrogel was made from a poly(*N*-isopropylacrylamide) (PNIPAAm) nanocomposite incorporating glycidyl methacrylate functionalized graphene oxide (GO-GMA). As a result, the nanocomposite hydrogel will undergo a large volume change under stimulation of infrared (IR) light, because of the highly efficient photo thermal conversion of GO-GMA. This material has the potential to be used as an actuator in microelectromechanical systems or microfluidic devices [35].

**Figure 2:**
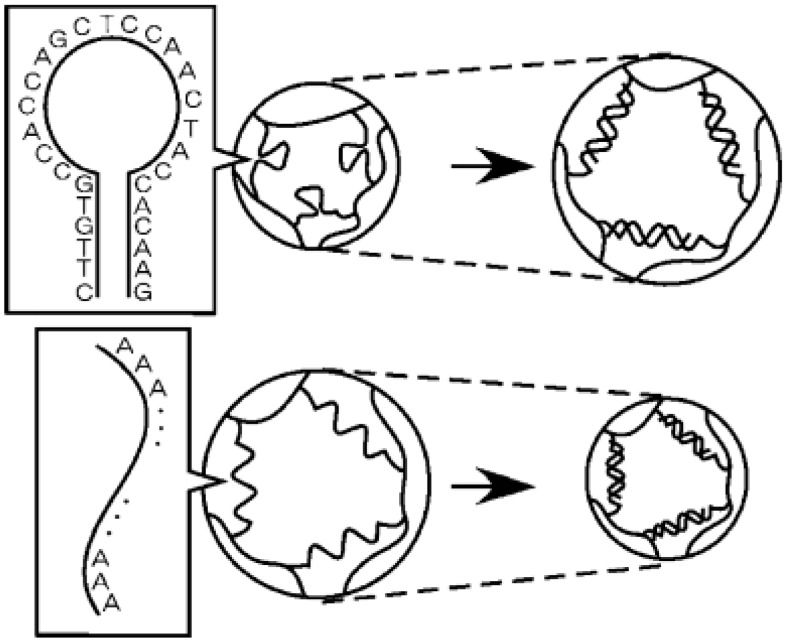
The response of novel hybrid hydrogels containing ssDNA as a cross-linker to ssDNA. Reprinted from [36] with permission from the American Chemical Society (2005).

In a different work done by Maeda’s group [36], they reported DNA-responsive hydrogels that “only shrunk” by the addition of ssDNA (single-stranded DNA) samples (Figure 2). This biomaterial was developed by polyacrylamide (polyAAm) hydrogels containing directly grafted ssDNA or an ssDNA-polyAAm conjugate in a semi-interpenetrating network. Unlike traditional stimuli-responsive hydrogels, this conjugate retains the advantage of using cross-linkable ssDNAs with well-characterized conformational properties, thus providing potential applications in DNA sensing or DNA-triggered actuators.

## 3. Different Theories behind the Hydrogel Swelling Mechanism

The properties of hydrogels for specific applications depend on their bulk structures. For network structure characterizations, there are several important parameters such as volume fraction in the swollen state, the corresponding mesh size, and the molecular weight of the polymer chain between neighboring crosslink points. The volume fraction of polymers in the swollen state is a parameter describing how much fluid can be absorbed and retained. The molecular weight between neighboring crosslink points, either covalent bond or physical interaction, is a parameter describing the degree of crosslinking. These parameters are related to each other and can be calculated theoretically or determined by a variety of experimental techniques. In the following paragraph, the two most widely used methods, the equilibrium swelling theory and the rubber elasticity theory, will be discussed.

### 3.1. Equilibrium Swelling Theory

The Flory–Rehner equation describes the mixing of polymers and liquid molecules, which can be used to analyze hydrogels without ionic domains [37]. The equilibrium status of the hydrogel swollen in a fluid is determined by two reverse forces. One is the thermodynmical force of mixing favors swelling, while the other is the stored force in the stretched polymer chains hindering swelling [30].

These two forces balance out each other as described in Equation (1) for the physical situation in terms of the Gibbs free energy:

(1) $$∆G_{\mathrm{total}}\;=\;∆G_{\mathrm{elastic}}\;+\;∆G_{\mathrm{mixing}}$$

where Δ*G*_elastic_ comes from the elastic stored forces in the extended polymer chains contained in the gel networks; Δ*G*_mixing_ is the result from the mixing between fluid molecules with the polymer chains. The mixing factor is a measure of the compatibility of the polymer with the solvent molecules, which is usually expressed by the polymer–solvent interaction parameter, χ [38].

Differentiation of Equation (1) with respect to the number of solvent molecules, while keeping the temperature and pressure constant, gives Equation (2):

(2) $$\mu_{1}{\;-\;\mu }_{1,o}\;=\;∆\mu_{\text{elastic}}\;+\;∆\mu_{\text{mixing}}.$$

In the equilibrium status, the chemical potential outside the gel should be equal to the chemical potential inside the gel (Δμ_1,o_ = Δμ_1_). As a result, the chemical potential change from free energy of mixing and elastic force stored in the stretched polymer chains have to cancel out each other.

The previous Flory–Rehner theory was modified for a hydrogel synthesized from aqueous phase. The water contained sufficiently changed chemical potential due to elastic forces, which is responsible for the change of volume fraction density of the polymer chains in the crosslinking process [39]. The presence of ionic moieties in hydrogel makes the situation much more complex, due to thermo complex system from the ionic domain of the polymer chains, which introduces an extra changing factor into the Gibbs free energy.

### 3.2. Rubber Elasticity Theory

From a mechanical perspective, hydrogels assemble natural rubbers that deform elastically in response to applied stress. Treloar [40] and Flory [41] utilized the elastic properties of the hydrogels to describe their structure. However, the original elasticity theory does not apply to hydrogels prepared in solvent. The theory of rubber elasticity by Peppas as in Equation (3) [42] is the only form used to analyze the hydrogel structure, with hydrogels prepared in solvent:

(3) $$\tau \;=\;\frac{\rho RT}{{\bar{M}}_{\text{c}}}(1\;-\;\frac{2{\bar{M}}_{\text{c}}}{{\bar{M}}_{\text{n}}})\;(\alpha \;-\;\frac{1}{\alpha^{2}}){\left(\frac{v_{2,s}}{v_{2,r}}\right)}^{1/3},$$

where τ is the applied stress to the polymer sample, ρ is the density of the polymer, *R* is the universal gas constant, *T* is the absolute experimental temperature, and *M*_c_ is the molecular weight between crosslinks. To utilize this elasticity theory to analyze the structure of the hydrogel, experiments must be done in the tensile mode [43,44].

### 3.3. Mechanism of Gelation

In the thermally induced sol–gel transition, there are different processes involved including hydrophobic and hydrophilic interactions, coil to helix transition, micelle packing, and so on. To understand the exact gelation mechanism behind certain polymers, figuring out the exact molecular level process is essential [45]. The most widely reported thermally induced gelation is based on the equilibrium between hydrophobic and hydrophilic interactions. For example, introducing a hydrophobic segment such as methyl, ethyl, or propyl to hydrophilic polymers is an efficient way to tune the hydrophobicity of the polymer [46]. LCST is a critical temperature below which the system will be miscible and above which phase separation will occur, forming gels. Interactions between polymer and polymer, polymer and water, and water and water take place in aqueous polymer solutions. The LCST of the system depends on the equilibrium status of these interactions. The most efficient way to determine LCST is by light scattering, with the collapse and aggregation of the polymer chains during gelation state inducing a dramatic increase of the light scattering [47].

Thermodynamically, the thermally induced abrupt change in the solubility is controlled by the Gibbs free energy of mixing [48]. A small change in temperature can cause negative change in the Gibbs free energy. As a result, the interaction between polymer and water will be eliminated and the water–water and polymer–polymer interaction will be favored. To equilibrate this negative Gibbs free energy change, there must be an increase in the entropy term due to the already known increased enthalpy term. Due to the dramatic increase in the hydrophobic interactions between polymer chains, at the sol–gel transition temperature, the polymer chains quickly dehydrate and collapse to a more hydrophobic structure [45,49]. On the other hand, some amphiphilic block copolymers will self-assemble into micelle structures due to the hydrophobic interaction to equilibrate the decrease of the Gibbs free energy [50].

Depending on the concentration, amphiphilic block copolymers can form micelles, which are aggregates of surfactant molecules dispersed in a liquid colloid and hydrogels by adding water and adjusting the temperature. These block copolymers build up a structure with a hydrophobic core and hydrophilic shell with typical micelle size between 20 and 100 nm. All the gelation mechanisms discussed here are based on reversible physical linkage, so the gelation transition is reversible after removing the gelling stimuli.

### 3.4. Calculation of the Mesh Size

The space contained in a hydrogel, responsible for the diffusion properties, is often regarded as the ‘pore’. Depending on the size of these pores, hydrogels are commonly classified as macro-pores, micro-porous, or non-porous. The size of the pore is often described by a structural parameter, the correlation length ξ, which is defined as the linear distance between two neighboring crosslinks [51]:

(4) $$\xi \;=\;\alpha {\left({{\bar{r}}_{o}}^{2}\right)}^{1/2}.$$

Here, α is the elongation ratio of the polymer chains and ${\bar{r}}_{o}$ stands for the distance between two adjacent crosslinking points of the unperturbed polymer chain [52]. From the volume fraction of the swollen polymer *v*_2,s_, the elongation ratio α can be calculated as:

(5) $${\alpha \;=\;v}_{2,s}^{-1/3}.$$

The unperturbed end-to-end distance of the polymer chain between two neighboring crosslinks can be calculated by:

(6) $${\left({{\bar{r}}_{o}}^{2}\right)}^{1/2}{\;=\;l(C}_{n}{N)}^{1/2},$$

where *l* is the length of the bond along the polymer backbone (1.54 Å for vinyl polymers), *C_n_* is the Flory characteristic ratio, and *N*, the number of links per chain, can be calculated by:

(7) $$N\;=\;\frac{2{\bar{M}}_{c}}{M_{r}},$$

where *M*_r_ is the molecular weight of the repeat unit. Finally, combining all the equations above, the correlated distance of the polymer chains between two adjacent crosslinking points can be evaluated:

(8) $$\xi \;=\;v_{2,s}^{-1/3}{\left(\frac{2C_{n}{\bar{M}}_{c}}{M_{r}}\right)}^{1/2}l.$$

## 4. Hydrogels Based on Natural Materials

### 4.1. Hydrogels Based on Polysaccharides

Cellulose is a natural polysaccharide that cannot dissolve in water. Unlike other types of water-soluble polysaccharides, cellulose requires separate cross-linking to fabricate a hydrogel network [53]. Native cellulose nanofibers are normally generated from bacterial and plants. Those nanofibers prefer to disperse in an aqueous solution rather than dissolving [54]. In Yliperttula and co-workers’ work [55], a plant-derived nanofibrillar cellulose (NFC) hydrogel with desired functionality makes a potential 3D cell culture scaffold. The structural properties of NFC hydrogel were evaluated along with rheological properties, cellular biocompatibility, cellular polarization, and differentiation of human hepatic cell lines. Due to its fluid-like property, NFC was demonstrated to be injectable at high stress, which makes it possible to mix into gels. Furthermore, spontaneous gelation after injection imparted the necessary mechanical support for both cell growth and differentiation.

Another technique was developed to immobilize an enzyme/antibody. After partial oxidation by sodium periodate, a cellulose hydrogel was prepared from an aqueous alkali-urea solvent. This enabled the cellulose gel to further introduce aldehyde groups [56]. By a Schiff base formation between the aldehyde and amino groups of protein, various active proteins can be covalently introduced to a cellulose gel and stabilized by a reduction of imines, which was confirmed by a coloring reaction. The same strategy is applicable to the peroxidase antibody, which makes various active proteins have the ability to be immobilized on cellulose gels by mild and facile processing. Due to the excellent chemical and mechanical stability of cellulose, this strategy and the afforded materials have the potential to be used for biochemical processing and sensing materials.

Reported by Lee and co-workers [57], by the use of a biocompatible ionic liquid, lipase from *Candida rugosa* was successfully trapped into various cellulose–biopolymer composite hydrogels. A biocompatible ionic liquid, 1-ethyl-3-methylimidazolium acetate, was used, which is known to be one of the best solvents for lignocellulosic materials among the ionic liquids (ILs). The lipase was successfully immobilized in various cellulose composite hydrogels, which is the first report that successfully entrapped an enzyme into non-derivatized cellulose–biopolymer composite hydrogels.

### 4.2. Hydrogels Based on Polypeptides

Gelatin is a denatured product of collagen, a mixture of peptides and proteins produced by partial hydrolysis of collagen extracted from the skin, bones, and connective tissues of animals, which is easily available, degradable, and demonstrates good biocompatibility in vivo. Also, gelatin retains cell-binding motifs such as arginylglycylaspartic acid (RGD) and matrix metalloproteinase (MMP)-sensitive degradation sites, which is the critical component in cell encapsulation [58,59].

Kao’s group [60] developed an easy strategy using cysteine to modify gelatin through a bifunctional PEG. In this way, free thio groups can be introduced to gelatin chains based on thiolated gelatin and poly(ethylene glycol) diacrylate. Varying concentration and ratio of precursor provides these crosslinked gelatin-based hydrogels with easy mechanical property modulation. In a 3D environment, gelatin crosslinking modality is crucial for long-term integrin binding sites as well as supporting cell attachment and proliferation by cell morphology and proliferation study.

In another study done by Melero-Martin’s group [61], they demonstrated that bioengineering human vascular networks inside methacrylated gelatin (GelMA) constructed in a liquid form could be injected into immunodeficient mice, followed by instantaneous crosslinking when exposed to UV light. A solution of GelMA containing human blood-derived endothelial colony-forming cells (ECFCs) and bone marrow-derived mesenchymal stem cells (MSCs) can be injected into the subcutaneous space of an immunodeficient mouse and then rapidly crosslinked with a controllable degree of GelMA through the exposure time to UV light. For future regenerative applications, which require the formation of functional vascular beds in vivo, GelMA is a good way to deliver vascular cells due to its injectable form before crosslinking.

In another example [62], a three-dimensional scaffold containing self-assembled polycaprolactone (PCL) sandwiched in a gelatin-chitosan hydrogel was developed for application as a biodegradable patch for surgical reconstruction of congenital heart defects; it contains a thin, self-assembled PCL core, intended to facilitate handling, cutting, and suturing the material and to provide sufficient tensile strength to function in the ventricular wall. The developed novel hydrogel was demonstrated to have significant potential to be used as a cardiac patch that can repair congenital cardiac defects.

## 5. Synthetic Hydrogels

Synthetic polymer-based hydrogels, due to their widely variable and easily tuned properties, have been extensively studied. By varying the chemical composition and preparation methods, the structure of the hydrogels can be controlled. Beneficial properties including porosity, swelling ability, stability, biocompatibility/biodegradability, and mechanical strength can all be tuned for specific application purposes. For example, vehicles with a controlled release rate for either small molecular or macromolecular drugs including DNA, enzymes, and peptides can be achieved [42].

Filipovic’s group [63] synthesized a novel temperature-and pH-sensitive hydrogel based on *N*-isopropylacrylamide (NIPAAm) and itaconic acid (IA) through free radical polymerization with lipase extracted from *Candida rugosa*, which is a promising system that can be applied as a pH-responsive device for drug delivery purposes. The properties of these prepared hydrogels were found to be highly sensitive to changes of temperature and pH, while keeping the ionic strength constant. A series of hydrogels was prepared with different molar ratios of NIPAAm and IA. Their morphology, mechanical properties, swelling degree, protein loading efficiency, and release rate were all evaluated. The protein release pattern clearly depends on the extent of swelling of the hydrogels.

Vuluga and co-workers reported the synthesis of a novel thermoresponsive crosslinked hydrogel based on different molecular weight of poly(propylene glycol)s (PPG) and diepoxy-terminated poly(ethylene glycol)s (PEG) to control the multi-block copolymer structure [64]. In an ideal situation, the hydrogel structure is expected to contain one PPG block and two PEG chains linked to the same amine group, leading to a structure with each PPG block surrounded by four PEG blocks, while each PEG block has two PPG blocks and two PEG blocks as neighbors. Both thermoresponsive and swelling properties can be adjusted by controlling the molecular weight of the constituent blocks or the salt added in.

For synthetic hydrogels, in addition to single network hydrogels, hydrogels consisting of two independently crosslinked polymer networks attracted wide attention recently, for which tough gels can be formed even with a less crosslinked “second work” within a more highly crosslinked “first network”. The molar ratio of the second network repeat units to the first network needs to be >5 [65].

In the work done by Spinks’s group [66], a novel double network hydrogel was synthesized with a bottlebrush structure formed from oligo-monomers of poly(ethylene glycol) methyl ether methacrylate as the first polymer network and poly(acrylic acid) as the second network. The strong intermolecular interactions between the neutral poly(ethylene glycol) side chains and the non-ionic groups offer the hydrogel excellent mechanical strength and high sensitivity to pH changes. Such a material with robust nature and sensitivity to pH changes has a potential as artificial muscles or controlled release devices.

In situ gelable interpenetrating double-network hydrogels have been formulated, prepared from thiolated chitosan and oxidized dextran in a one-pot process. No potentially cytotoxic small-molecule cross-linkers are required and they are without complex maneuvers or catalysis. It was reported that the interpenetrating double-network structure enhanced the mechanical properties and gelation performance of the hydrogel formulated from the thiolated chitosan. In conclusion, this hydrogel system is promising as an in situ formable biomaterial for clinically related applications requiring mechanical strength, durability, and fast gelation [67].

A new inorganic–organic double-network hydrogel composing of poly(acrylic acid) and graphene was prepared with the as-prepared 3D graphene architecture to be the first network and acrylic acid monomer dispersed into the consecutive channels and polymerized which is the second network. This inorganic–organic double-network hydrogel shows both flexibility and electrical conductivity, and can be used in the next generation of flexible electric devices [68].

## 6. Applications in Biomedical Field

### 6.1. Hydrogels for Three-Dimensional Cell Culture

**Figure 3:**
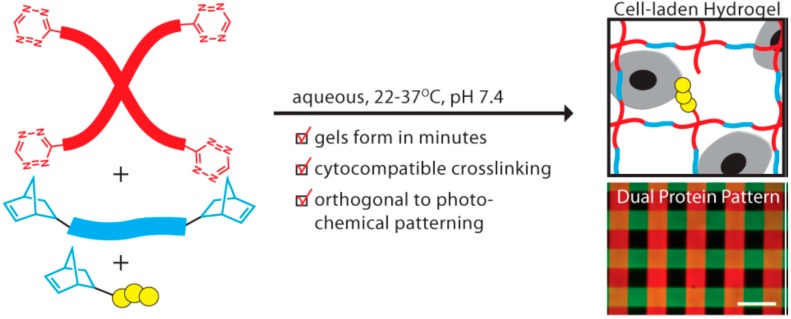
Synthetically tractable click hydrogels for three-dimensional cell culture formed using tetrazine–norbornene chemistry. Reprinted from [71] with permission from the American Chemical Society (2013).

Hydrogels, with high water content as well as tissue-like mechanical properties, have been demonstrated to be capable of combining with cells to engineer various tissues in both vitro and vivo [69,70]. A crucial requirement for the construction of three-dimensional regenerative tissue in sufficient quantities is an artificially created environment that enables biological cells to grow or interact with their surroundings in all three dimensions. Anseth’s group [71] reported a new cross-linking chemistry by the tetrazine-norbornene click reaction for the formation of cell-laden hydrogels for 3D cell culture (Figure 3). A PEG functionalized with benzylamino tetrazine moiety was specifically chosen and used because in their previous work it was shown to have high reactivity toward norbornene. The bio-orthogonality, ideal reaction kinetics, and amenability to photochemical patterning made this hydrogel platform have potential applications in a variety of fundamental as well as translational tissue engineering applications.

Another work was done by Loessner and co-workers [72]. They characterized gelatin methacrylamide (GelMA)-based hydrogels and established them as in vitro and in vivo spheroid-based models for ovarian cancer, to efficiently reflect the advanced disease stage of patients. Hydrogels of equal size, diffusion, and physical properties were generated by employing a controlled preparation and validation protocol. Such GelMA-based hydrogels served as a low-cost, reproducible, and tailorable matrix for 3D cancer cell cultures. Thus, they can be applied as an alternative to improve the understanding of disease progression on a cellular level and also to screen anti-cancer drugs.

### 6.2. Hydrogels for Self-Healing

Self-healing is one of the most outstanding properties of natural materials such as skin, bones, and wood. Thus, hydrogels that can self-heal open up another field for biomedical applications [73,74]. Even though synthetic hydrogels are fabricated to mimic biological tissues, most of the time they still lack the ability to self-heal. This drawback will limit their utilization in many applications that require high stress. As a consequence, researchers dedicate a lot of effort to improving the mechanical properties of hydrogels, including the self-healing property.

The process of healing cracks in natural systems usually contains an energy dissipation mechanism. The self-healing can occur in the presence of sacrificial bonds, which can break and reform dynamically before or during the failure occurring. To prepare a self-healing hydrogel, both covalent [75,76] and non-covalent [77,78] interactions have been reported.

The synthesis of novel hydrogels containing reversible oxime crosslinks were reported by Mukherjee [79]; they are capable of autonomous healing as a consequence of their dynamic nature. To prepare these hydrogels, copolymers containing keto functional groups were synthesized by copolymerizing *N*,*N*-dimethylacrylamide (DMA) and diacetone acrylamide (DAA) via free radical polymerization. Then the afforded hydrophilic copolymers were covalently crosslinked with difunctional alkoxyamine to obtain hydrogels by the formation of oxime. Along with the efficient self-healing ability, the reversibility of oxime linkages also led to reversible gel-to-sol transitions when excess monofunctional alkoxyamine was added at ambient temperature.

Other than chemical cross-linkers, hydrophobic interactions can also play an important role as a cross-linker for self-healing hydrogels. Okay’s group developed a hydrogel by copolymerization of a large hydrophobic monomer stearyl methacrylate and dococyl acrylate with a hydrophilic monomer acrylamide in a micellar solution of sodium dodecyl sulfate [80]. After the addition of salt, micelles grow and solubilize hydrophobes. This hydrogel was demonstrated to have a high degree of toughness due to the finite lifetime of hydrophobic interactions between stearyl methacrylate and dococyl acrylate blocks.

**Figure 4:**
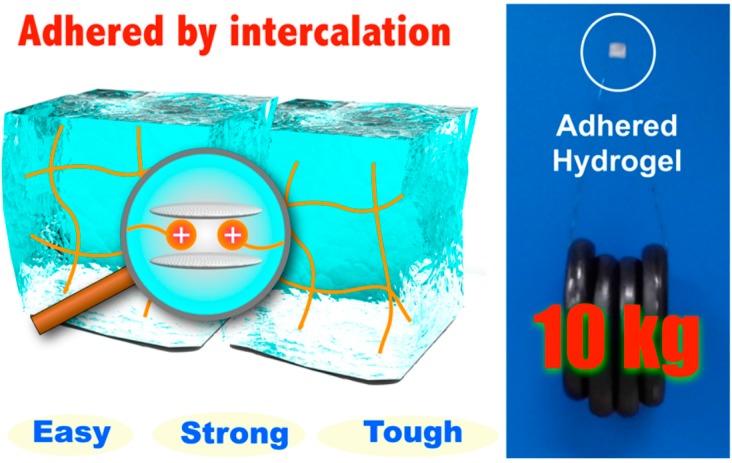
Schematic representation of high concentration cationic gels adhered using 1.6 mg of Micromica supporting a tensile load of 10 kg. Reprinted from [81] with permission from the American Chemical Society (2016).

A different work was recently reported by Yamauchi and co-workers [81]. In this research, hydrogels containing cationic substituents were prepared via free-radical polymerization. After applying an aqueous dispersion of Micaromica to the surface, when brought into contact the hydrogels adhered strongly due to the intercalation of cationic substituents included in the gel networks into the interlayers of Micromica. The adhesive strength became higher and was able to support a tensile load of 10 kg as the water content ratio of hydrogels decreased (Figure 4).

### 6.3. Hydrogels for Drug Delivery

To deliver drugs, porous structure of hydrogels can provide a matrix for drug loading and protect drugs from hostile environment at the same time. Moreover, this porosity can be controlled by varying the crosslinking density of the gel matrix. The release rate, another important parameter for drug carriers, mainly depends on the diffusion coefficient of this molecule through the gel network and can also be tuned according to specific requirements. Biocompatibility and biodegradability can be obtained by designing certain chemical and physical structures for hydrogels. All of those properties lend hydrogels great potential to be used for drug delivery [46,82].

Poly (ethylene oxide)-*b*-poly(propylene oxide)-*b*-poly (ethylene oxide) triblock copolymers (PEO–PPO–PEO) (known as Pluronic or Poloxamer) have been extensively used in pharmaceutical systems [83]. Paavola and co-workers fabricated an injectable gel based on Poloxamer to carry and control the release of the anesthetic agent lidocaine [84]. Since Poloxamer is commercially available, this method was proven to be suitable for hospital utilization. Nevertheless, the relatively rapid diffusion of drugs out of the gel matrix, as well as the duration of drug release, is still limited. It can be improved by covalent crosslinking with other functional group, such as ethoxysilane, amine, or carbohydrates, to prevent the dilution of the polymer in water [85,86,87].

In addition, to load drug into a gel matrix, conjugating drugs to a hydrogel crosslinked network is another way to deliver drugs. Zhu and co-workers reported a supramolecular based hydrogel to deliver doxorubicin (DOX). First, they synthesized a supramolecular polymeric prodrug via host–guest interaction between cyclodextrin-functionalized polyaldehyde and DOX-modified adamantine. After crosslinking by carboxymethyl chitosan, an injectable DOX-loaded hydrogel was created. This hydrogel was shown to release DOX when exposed to acid stimuli [88].

## 7. Conclusions

Compared with other types of biomaterials, hydrogels have distinct properties such as high water content, controllable swelling behavior, ease of handing, as well as biocompatibility, which makes them attractive for biomedical applications. Based on their chemical structure and crosslink network, hydrogels can respond to different types of stimuli including thermal, pH, light, and chemical stimuli, which can meet various application requirements. Two different hydrogel swelling mechanisms were discussed to give a thorough understanding of how the bulky structure affects the properties of the swollen hydrogels under specific conditions. Hydrogels based on natural materials such as polysaccharides and polypeptides, along with synthetic hydrogels were exampled in detail. Hydrogels, which are three-dimensional crosslinked polymeric networks able to swell in large amounts of water, should be considered prime candidates for carriers or matrices for cells in tissue engineering, self-healing materials, and delivery vehicles for drugs and biomolecules. Further research should be directed to achieving high mechanical strength, fast and efficient self-healing ability, and various biological activities for different biomedical purposes.
